# Targeting ASK1 by CS17919 alleviates kidney‐ and liver‐related diseases in murine models

**DOI:** 10.1002/ame2.12437

**Published:** 2024-06-14

**Authors:** Guoqiang Liao, Qianjiao Yang, Xuhua Mao, Yiru Zhao, Beizhong Chen, Kun Zhang, Yu Zhang, Ping Zhang, Zhengli Chen, Shengjian Huang

**Affiliations:** ^1^ Chengdu Chipscreen Pharmaceutical Corp., Ltd. Chengdu Sichuan P.R. China; ^2^ Laboratory of Experimental Animal Disease Model College of Veterinary Medicine, Sichuan Agricultural University Chengdu Sichuan P.R. China; ^3^ Shenzhen Chipscreen Biosciences Co., Ltd. Shenzhen Guangdong P.R. China

**Keywords:** ASK1_1_, CKD_4_, CS17919_2_, GS‐4997_3_, NASH_5_

## Abstract

**Background:**

Apoptosis signal‐regulating kinase 1 (ASK1) is a MAP3K kinase in the MAPK signaling pathway activated by stressors and triggers downstream biological effects such as inflammation and apoptosis; therefore, inhibition of ASK1 kinase activity can protect cells from pathological injury. In this study, we designed and synthesized a novel selective ASK1 inhibitor, CS17919, and investigated its pharmacological effects in various animal models of metabolic injury.

**Methods:**

First, we validated the ability of CS17919 to inhibit ASK1 in vitro and then tested the safety profile of CS17919 in cell lines compared with Selonsertib (GS‐4997), a phase III ASK1 inhibitor. We then conducted pharmacokinetic (PK) studies in mice. Finally, we tested the in vivo efficacy of CS17919 in murine models of chronic kidney disease (CKD) and non‐alcoholic steatohepatitis (NASH).

**Results:**

Compared to GS‐4997, CS17919 demonstrated comparable inhibition of ASK1 in vitro, exhibited lower toxicity, and provided greater protection in palmitic acid‐treated LO2 cells. CS17919 also showed pronounced pharmacokinetic properties such as a high plasma concentration. In the unilateral ureteral obstruction model (UUO), CS17919 and GS‐4997 preserved kidney function and showed a non‐significant tendency to alleviate kidney fibrosis. In the diabetic kidney disease (DKD) model, CS17919 significantly improved serum creatinine and glomerular sclerosis. In the NASH model, the combination of CS17919 and a THRβ agonist (CS27109) was found to significantly improve liver inflammation and substantially reduced liver fibrosis.

**Conclusions:**

CS17919 showed cell protective, anti‐inflammatory, and antifibrotic effects in vitro and in vivo, suggesting its therapeutic potential for metabolic‐related kidney and liver diseases.

## Graphical abstract

Unmet clinical need in chronic kidney disease and NAFLD. Inhibition of ASK1 improves inflammation and fibrosis. Animal models can mimic disease progression.

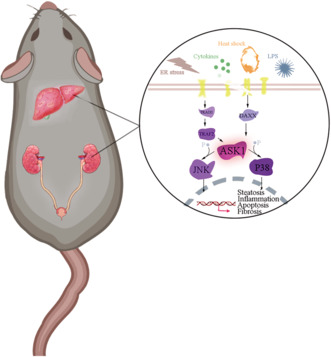

## 1 INTRODUCTION

Mitogen‐activated protein kinase (MAPK) plays an important role in signal transduction and is activated by different extracellular stimuli such as cytokines, neurotransmitters, hormones, cellular stress, cell adhesion, etc. The MAPK pathway is conserved in eukaryotes from yeast to humans, including three kinase families, MAPK kinase kinase (MAPKKK), MAPK kinase (MAPKK) and MAPK, which are activated sequentially and collectively to regulate cell growth, differentiation, adaptation to environmental stress, inflammatory response, and many other vital physiological/pathological processes of the organism.^1^ Apoptosis signal‐regulating kinase (ASK) is a member of the MAPKKK family, which is constitutively expressed and participates in stress responses such as inflammation and apoptosis through ASK‐MKK‐JNK/P38 cascade signaling.^2^ The ASK family contains three kinases, ASK1, ASK2, and ASK3, in which ASK1 and ASK2 primarily mediate inflammation, while ASK3 mainly senses and responds to changes in osmotic pressure.^3^, ^4^ The c‐terminal helix–helix (CCC) domain of ASK1 homodimerizes to form a high molecular weight complex (the ASK1 signalosome) that remains active, and upon stimulation this complex grows in size to form a higher‐quality complex that activates ASK1.^5^ Human ASK1 consists of 1374 amino acids and has been extensively studied for its role mostly as a homodimer or heterodimer of ASK2.^6^ The homodimer forms a complex with thiol‐containing antioxidant proteins (Trx), glutoxigenin (Grx), and scaffolding proteins that keeps it in an inactive state. Upon stimulation by activating factors, Trx, Grx, or the scaffold proteins dissociate, and threonine at position 838 is autophosphorylated within the activation loop, thus completing the process of activating ASK1 and eventually activating P38/JNK.^7^, ^8^, ^9^

ASK1 is activated by a variety of factors including oxidative stress, endoplasmic reticulum (ER) stress, heat shock, inflammatory cytokines, and lipopolysaccharide (LPS), and is then phosphorylated and dimerized to trigger a complex and diverse range of downstream biological effects. ASK1 is widely expressed in the liver and kidney, and its sustained overactivation results in inflammation and apoptosis that often causes severe damage to the organism. In the liver, ASK1 activation mediates steatosis, inflammation, and cell death, which contribute to the development of NASH, a worldwide disease with no approved medications as yet. ASK1 activation is also correlated with multiple stimulators in NASH progression, such as tumor necrosis factor (TNF) receptor and G protein‐coupled receptor (GPCR).^10^, ^11^ The ASK1‐JNK‐p38 pathway was found to be overactivated in several animal models of NASH,^12^, ^13^ and blocking ASK1 N‐terminal dimerization and autophosphorylation with a short CFLAR peptide or administering the ASK1 inhibitor GS‐444217 significantly ameliorated NASH symptoms.^14^, ^15^ In clinical studies, hyperactivation of ASK1 has been positively associated with insulin resistance, inflammation and steatosis.^16^, ^17^ Overactivated ASK1 is also found in other liver‐related diseases, such as diabetes and hyperlipidemia.^18^, ^19^ Similarly, hyperactivation of ASK1, together with activation of P38, downstream of ASK1, is also observed in kidney‐related conditions, such as acute and chronic kidney injuries. The massive activation of ASK1 in glomerular, peritubular capillary, and tubular epithelial cells after renal transplantation is not seen in normal kidneys,^20^ and p‐ASK1, p‐p38 MAPK, p‐JNK have been positively correlated with renal injury in acute renal ischemia and UUO models.^21^ Diabetic nephropathy, or diabetic kidney disease (DKD), is a typical chronic kidney injury disease in which diabetes‐induced oxidative stress drives p38 activation, and ASK1 is a major contributor in this process, as confirmed by both human and animal studies.^22^, ^23^ Intriguingly, mice with ASK1 knockout develop normally and functionally, and their liver and kidneys do not show significant inflammation and fibrosis when exposed to the same stimulus. This motivated us to target ASK1 inhibition as a therapeutic strategy to treat diseases such as NASH and DKD.

Consequently, we designed a new ASK1 inhibitor, CS17919 (Figure S1A), and evaluated its efficacy in vitro and in vivo. Our results showed that CS17919 inhibited ASK1 activity at the nanomolar level in vitro and improved kidney and liver injury in vivo. This study indicates that targeting ASK1 via CS17919 is a potential strategy to treat diseases with unmet clinical needs such as DKD and NASH.

## 2 METHODS

### 2.1 Materials

Both CS17919 and CS27109 were designed and synthesized (with a purity over 98%) by Shenzhen Chipscreen Biosciences Co., Ltd (Shenzhen, China). GS‐4997 was purchased from MedChemExpress (Monmouth Junction, NJ, USA). CS17919 and GS‐4997 were dissolved in sterile DMSO for the in vitro study. For in vivo administration, both CS17919 and CS27109 were suspended in a solvent containing CMC‐Na (0.2%) and Tween‐80 (0.1%) for oral gavage and GS‐4997 was solubilized using 10% DMSO and 90% corn oil, followed by oral gavage. Cell lines were purchased from Nanjing KEBAI Biotechnology Co. (Nanjing, China) and palmitic acid (PA) was purchased from Sigma (P0500, St Louis, MO, USA). Cell Titer‐Glo for cell viability assays and MTS reagents for cytotoxicity assays were purchased from Promega (G9241 Madison, WI, USA) and Abcam (ab197010, Cambridge, UK), respectively. Hematoxylin & Eosin (H&E), Sirius Red, Masson, and PAS stains were purchased from Beijing Solarbio life sciences (G1120, G1472 and G1281, Beijing, China).

### 2.2 Enzymatic activity analysis

The inhibitory activity of CS17919 and GS‐4997 against the human ASK1 kinase was determined by a homogeneous time resolved fluorescence (HTRF) assay using a full‐length recombinant human ASK1 protein (M073107) (Cisbio, Codolet, France).^24^ Buffer and reagent preparation and assay protocol are presented separately in the Table S1. Enzymatic activity analysis was repeated three times. IC_50_ results were obtained using Prism 8.0 (GraphPad Prism Software).

### 2.3 Cell viability and cytoxicity assay

L02 cells were cultured in DMEM medium (Gibco, 11 965 092, Carlsbad, CA, USA) supplemented with 20% FBS and 1× penicillin–streptomycin. L02 cells were inoculated in two 96‐well plates at 1E4 cells/well (200 μL). The following day, the cells were stimulated with 50 μmol/L PA (dissolved in ethanol to make a 200× stock solution and then added to the medium) and simultaneously incubated with different concentrations of CS17919 and GS‐4997 (CS17919 and GS‐4997 stock solutions were added to the well plates to reach a final concentration of 10–0.3 μmol/L). After 72 h of incubation, the original medium was removed, followed by the addition of 100 μL of new DMEM medium and 60 μL of Cell Titer‐Glo reagent per well. The cells were incubated for 5 min in the dark, and then transferred to a black permeable plate for detection using an enzyme plate reader (Tecan spark, Grödig, Austria).

Human embryonic kidney cells (HEK‐293A), human hepatocellular carcinoma cells (HUH7), human embryonic liver diploid cells (CCC‐HEL‐1) and human embryonic lung cells (MRC‐5) were selected for the cytotoxicity study of both drugs, using the MTS assay protocol. Cells were cultured at 37°C with 5% CO_2_ using an appropriate medium. For the assay, the cells were evenly distributed at 1E4 cells/well in 96‐well plates, and then CS17919 or GS‐4997 was added to the plates in a dose‐dependent manner (3‐fold dilution from 100 μmol/L). After 72 h of incubation, 30 μL of MTS solution was added to each well, and the cells were then incubated for 2–4 h in the dark. The absorbance value of each well at 490 nm was measured using an enzymatic plate reader and the IC_50_ for each drug and each cell line was calculated. Cell viability and cytoxicity experiments were repeated three times.

### 2.4 Pharmacokinetic study

Male C57BL/6J mice (Beijing Huafukang Biotechnology Co., Ltd, Beijing, China) were selected at 6–8 weeks for the experiment. A single gavage of CS17919 was performed according to body weight. About 50–60 μL of blood and tissue were isolated in anticoagulation tubes at 15, 30 min, 1, 2, 4, 6, and 8 h after administration, respectively, and then centrifuged at 5000 r/min for 7 min at 4°C. The plasma and tissue samples were fully shaken, and the standard curve and lower limit of quantification were determined according to the compound response values (standard curves were established during the analysis of each sample). The standard solution was prepared with 7 concentrations and QC with 3. The standard and QC working solutions were added to the 96‐well plate, and the protein was precipitated by adding acetonitrile solution containing the internal standard together with the sample to be tested. After shaking for 10 min and centrifugation at 12 000 × *g* for 10 min, the supernatants from the 96‐well plate were analyzed by LC–MS (mobile phase: 0.1% formic acid‐water 0.1% formic acid‐acetonitrile). Raw data were imported into Phoenix WinNonlin to calculate AUC, *C*_max_, *T*_1/2_, etc.

Male C57BL/6J mice at 6–8 weeks (Beijing Huafukang Biotechnology Co., Ltd) were also selected for tissue distribution studies. Mice were gavage‐administered at 20 mg/kg and anesthetized at 1, 2, 4, and 8 h after administration for tissue collection, including whole blood, heart, liver, kidney, and brain. These organs were homogenized using a tissue homogenizer and analyzed together with the plasma according to the standard LC/MS procedure.

### 2.5 Animal studies

All animal experiments were performed according to the guidelines for the care and use of animals approved by Chengdu Chipscreen Pharmaceutical CORP., Ltd Council (Chengdu, China). All animals were housed in a facility with a temperature of 20–26°C, a humidity of 40%–70%, a 12/12 h light/dark cycle and free access to food and water.

#### 2.5.1 UUO model

55 C57BL/6J mice, male, 6–8 weeks old were purchased from Chengdu Pharmachem Biotechnology Co. (Chengdu, China) and randomly divided into 5 groups according to their body weight: Sham (*n* = 10), Model (*n* = 15), CS17919L (10 mg/kg, *n* = 10), CS17919H (60 mg/kg, *n* = 10), and GS‐4997 (30 mg/kg, *n* = 10). The mice were anesthetized and fixed on the operating table, and the abdominal cortex and muscles were cut in the middle of the right side of the abdomen, and the ureter was searched for along the renal hilum. The ureter was separated using a glass split‐needle and ligated at the opening of the renal pelvis and the upper 1/3 of the ureter, and the ureter was cut between the two ligatures. Finally, the muscle and skin were closed with consecutive sutures, and animals were kept under observation until they awoke. Both the Sham and the Model group received solvent (0.2% CMC‐Na + 0.1% Tween‐80) orally twice daily. CS17919 and GS‐4997 were administered by gavage twice daily at the corresponding doses for 11 days. All treatments were started one day before surgical operation. All mice were sacrificed one day after administration with overnight fasting. Blood was collected, and serum was isolated for the analysis of urea nitrogen and creatinine. The kidney of each mouse was removed, weighed and fixed in 10% formalin solution for pathological study.

#### 2.5.2 DKD model

26 male neonatal mice were purchased from Chengdu Pharmachem Biotechnology Co., 5 of which were assigned to the control group and fed a chow diet. The other 21 mice were subcutaneously injected with STZ (100 mg/mouse) 48 h after birth. After 4 weeks, these mice were fed a high‐fat diet (HFD, Research Diet, New Brunswick, NJ, USA) and randomly subdivided into two groups based on blood glucose level followed by 6 h of fasting: Model (*n* = 11) and CS17919 (*n* = 10). Both the control and model groups were given the solvent mentioned above, administered orally twice a day, while CS17919 was administered twice a day by gavage at a dose of 50 mg/kg for 70 days. Body weight measurements were performed twice a week during the administration period. 24‐h urine was collected for each mouse 2 days before sacrifice. Creatinine clearance was calculated using the following formula: creatinine clearance = urinary creatinine (mg/dL) × urine volume per minute (ml/min)/blood creatinine (mg/dL). Fasting blood glucose was measured before sacrifice. After a 10‐week drug intervention, mice were sacrificed, and blood was collected to isolate serum for the urea nitrogen and serum creatinine test. The kidneys of each mouse were then removed, weighed and fixed in 10% formalin solution for pathological study.

#### 2.5.3 NASH model

41 C57BL/6J mice, male, 6–8 weeks of age, were purchased from Chengdu Dossy Experimental Animal Co. (Chengdu, China), 9 of which were assigned in the control group and fed a chow diet. The other 32 mice were fed a HFD (D12429, Research diet, USA). After 8 weeks of feeding, these mice with HFD were subcutaneously injected with CCL_4_ (0.1 mL/kg) twice a week. These mice were then randomly subdivided into 4 groups according to serum ALT and AST at week 12: Model (*n* = 8), CS17919 (*n* = 8), CS27109 (*n* = 8), and Combination (*n* = 8). Both the control and model groups were given the solvent mentioned above, administered orally twice a day, while CS17919 was administered twice a day by gavage at a dose of 20 mg/kg and CS27109 was administered twice a day by gavage at a dose of 10 mg/kg for 4 weeks. The body weight of each mouse was measured every 3 days during the drug intervention. All animals were fasted overnight after the last drug administration and blood was collected to isolate serum for biochemical tests including AST, ALT, TG, TC, HDL‐c, and LDL‐c. The liver of each mouse was removed, the right lobe of which was fixed in 10% formalin solution for pathological study.

### 2.6 Histopathological studies

#### 2.6.1 H&E staining

H&E staining was applied to determine scores for liver steatosis, ballooning and inflammation in the NASH model. Thin 4 μm paraffin sections of each sample were used to assess liver histology, following a standard protocol. Paraffin sections were deparaffinized and placed in Hematoxylin staining solution for 3–8 min before using tap water to elute excess Hematoxylin. Sections were stained in Eosin stain for about 1–3 min and rinsed with tap water. Finally, the slices were dehydrated and sealed. Score ranges were 0–3 for liver steatosis, 0–2 for ballooning and 0–12 for inflammation (which included scores of 0–3 for portal inflammation, 0–3 for lobular inflammation, 0–3 for piecemeal necrosis, and 0–3 for bridging necrosis).

#### 2.6.2 Masson staining

Masson staining was applied to determine kidney fibrosis in the UUO model. Thin 4 μm paraffin sections of each sample were used in this study, which followed the standard procedure. The sections were stained with Hematoxylin staining solution for 5–10 min and then washed, followed by staining with Ponceau S‐acid fuchsin stain for around 5–10 min. The sections were treated with 1% phosphomolybdic acid and Aniline Blue solution, and finally dehydrated stepwise in alcohol and sealed. VIS7.0 software was used to analyze the area of kidney fibrosis and the percentage of the fibrosis area in the scanning area was calculated.

#### 2.6.3 PAS staining

PAS staining was applied to determine kidney glomerular sclerosis in the DKD model. Thin 4 μm paraffin sections of each sample were used in this study, which followed the standard procedure. Periodic acid‐treated paraffin sections were stained using Schiff for 10 min, after rinsing the residual acid. Hematoxylin staining was performed for 3 min, and the samples were then dehydrated and sealed. The PAS score was calculated by semiquantitative analysis as follows: 0, without sclerosis; 1, 1%–25% sclerosis area; 2, 26%–50% sclerosis area; 3, 51%–75% sclerosis area; 4, >75% sclerosis area.

#### 2.6.4 Sirius Red staining

Sirius Red staining was applied to determine liver fibrosis in the NASH model. Thin 4 μm paraffin sections of each sample were used in this study followed the standard procedure. Sections were first stained with Hematoxylin for 5–10 min, then washed and stained with Sirius Red stain about 15–30 min. Finally, the sections were rinsed and sealed. Image‐Pro Plus 6.0 was used for analyzing the area of liver fibrosis and the percentage of the fibrosis area in each section was calculated.

### 2.7 Statistical analysis

All data from the experiment were counted using GraphPad Prism 8.0 software and expressed as mean ± standard deviation (SD). Student's t test was used to compare means between two groups and one‐way ANOVA was used among three or more groups. We chose the Tukey test if the assumption of homogeneity of variances was met. If the assumption of homogeneity of variances was not met, then the Dunnett's T3 test was recommended. *p* < 0.05 was considered statistically significant.

## 3 RESULTS

### 3.1 CS17919 showed pronounced inhibition of ASK1 and cell protection in vitro

**FIGURE 1:**
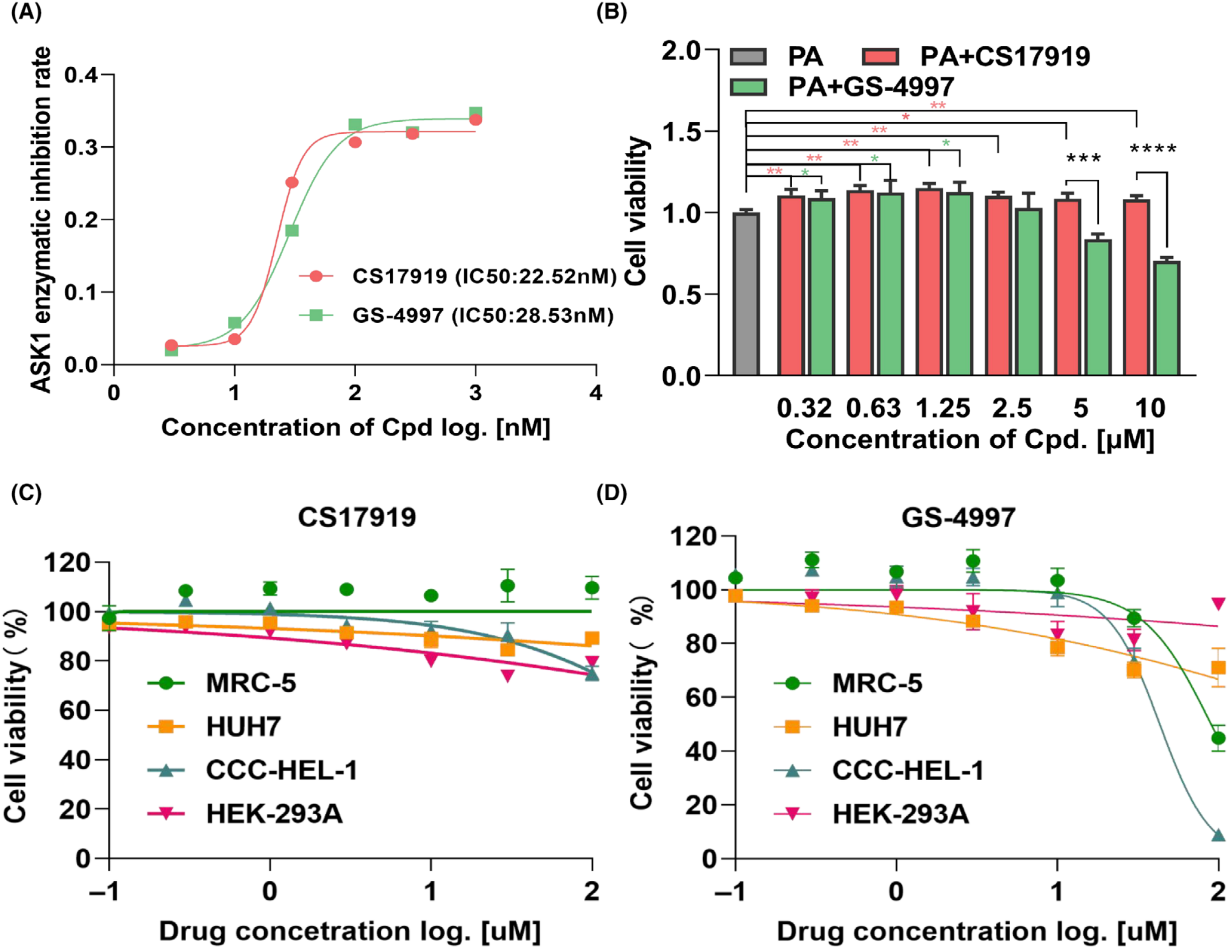
ASK1 inhibition, cell protection, and cytotoxicity of CS17919. (A), Inhibition of ASK1 kinase by CS17919 and GS‐4997 was determined by a HTRF assay at different concentrations (3–1000 nmol/L, with a 3.3‐fold dilution). (B), Cell protection of CS17919 and GS‐4997 was assessed in L02 cells stimulated by 50 μmol/L PA at different drug concentrations (0.32–10 μmol/L, with a 2‐fold dilution). Cytotoxicity assay of CS17919 (C) and GS‐4997 (D) was conducted in 4 different cell lines (MRC‐5, HUH7, CCC‐HEL‐1, and HEK‐293A) at different drug concentrations (0.3–10 μmol/L, with a 3.3‐fold dilution). *p < 0.05, **p < 0.01, ***p < 0.001, ****p < 0.0001 were obtained by one‐way ANOVA (post hoc by Tukey).

We performed an enzymatic assay to determine the inhibition of ASK1 by CS17919 compared with GS‐4997, a Phase III ASK1 inhibitor in the treatment of advanced NASH and DKD. CS17919 showed robust inhibition of ASK1 in a dose‐dependent manner with an IC_50_ of 22.52 nmol/L, which was equivalent to the IC_50_ of GS‐4997, at 28.53 nmol/L (Figure 1A).

Inhibition of ASK1 has been reported to protect cells from apoptosis and necrosis, which are frequently observed in organ injuries, such as in the liver and kidney. To validate this, we induced cell death of L02 cells by PA and treated them with CS17919 or GS‐4997 simultaneously to examine the protective effect of both drugs. First, we identified a concentration of 50 μmol/L PA for cell stimulation (Figure S1B), and then the cell viability of all treated samples was normalized to cells treated with PA. This showed that CS17919 significantly prevented apoptosis at all doses while GS‐4997 only protected against cell death at lower doses (Figure 1B). Surprisingly, at 5 and 10 μmol/L GS‐4997 reduced cell viability in a dose‐dependent manner, while CS17919 exhibited a mild protective effect at both doses. However, no significant differences in protective effect were observed between these two compounds at lower doses. This result indicated that CS17919 had a promising protective effect and a favorable safety profile.

Safety is a critical characteristic of a drug, especially for the treatment of chronic diseases. We observed that CS17919 was safer than GS‐4997 in L02 cells, as mentioned above (Figure S1C), but more work needs to be done to confirm this observation. To further confirm this, we performed a cytotoxicity assay of both CS17919 and GS‐4997 in 4 cell lines originating from the liver, kidney, and lung. As expected, CS17919 barely affected cell viability in the 4 cell lines (Figure 1C). However, GS‐4997 reduced cell viability at higher doses in CCC‐HEL‐1 and MRC‐5 cells, derived from human embryonic liver and lung, respectively (Figure 1D). Collectively, our results suggested that CS17919 might have a safer profile than GS‐4997 under the same in vitro conditions.

### 3.2 CS17919 exhibited favorable pharmacokinetics (PK) properties

We then carried out the PK study in mice at two doses, 20 mg/kg and 50 mg/kg, given orally. The result is shown in Table 1. CS17919 at both doses exhibited favorable PK properties and was dose dependent. However, the T1/2 of CS17919 is relatively short, which motivated us to increase the frequency of drug administration. We then carried out a tissue distribution experiment for CS17919 to determine the drug concentrations in different tissues at different time points. The result is shown in Table 2. Generally, tissue‐specific accumulation of CS17919 in the liver and kidney prompted us to evaluate its in vivo efficacy in liver or kidney related diseases.

**TABLE 1:** Pharmacokinetic parameters of CS17919 in C57BL/6J following oral administration.

Parameters	20 mg/kg. p.o.	50 mg/kg. p.o.
AUC_0–*∞*_ (h*ng/mL)	23 113 ± 5898.47	58 795 ± 23 199.32
AUC_0–*t*_ (h*ng/mL)	23 078 ± 5892.78	57 239 ± 20 606.29
C_max_ (ng/mL)	4039 ± 646.90	5741 ± 1835
MRT_0–*∞*_ (h)	3.96 ± 0.91	6.43 ± 1.44
*T*_1/2_ (h)	2.20 ± 0.74	3.50 ± 1.86
*T*_max_ (h)	1.50 ± 0.87	2.00 ± 0

*Note*: Data were shown as mean ± SD, *n* = 3.

**TABLE 2:** Tissue distribution of CS17919 in C57BL/6J following oral administration.

Organ	1 h	2 h	4 h	8 h
Heart (ng/g)	1330.94 ± 384.66	918.02 ± 156.73	338.03 ± 97.76	256.60 ± 117.57
Liver (ng/g)	8933.89 ± 456.44	5322.41 ± 114.54	2371.88 ± 421.50	1939.43 ± 337.32
Kidney (ng/g)	4353.18 ± 260.20	2751.58 ± 252.41	1173.69 ± 135.53	993.13 ± 136.67
Brain (ng/g)	30.42 ± 6.82	7.37 ± 1.98	8.28 ± 4.78	LLOQ (<1)
Plasma (ng/mL)	3104.56 ± 474.21	2737.71 ± 208.45	1444.51 ± 155.53	856.1 ± 332.34

*Note*: Data were shown as mean ± SD, *n* = 3. Brain tissue samples less than 1 ng/g (lower limit of quantification, LLOQ) at 8 h.

### 3.3 CS17919 improved kidney injury and fibrosis in the UUO model

**FIGURE 2:**
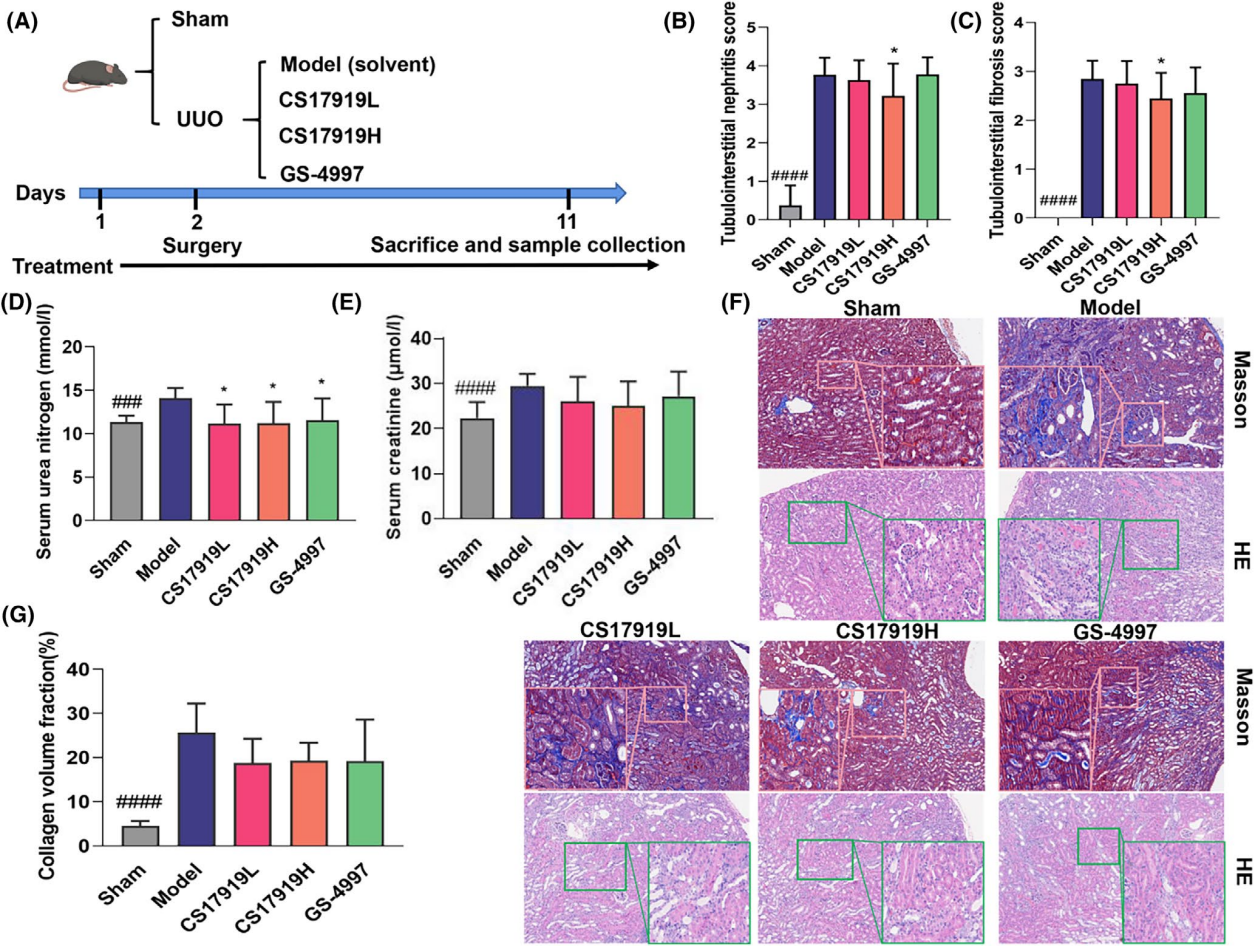
Evaluation of CS17919 efficacy in a UUO mouse model. (A), Schematic representation of CS17919 study in the UUO model. Tubulointerstitial nephritis scores (B) and tubulointerstitial fibrosis scores (C) were measured based on H&E staining. (D), Histological changes were assessed by Masson staining and H&E staining. Scale bar: 250 μm. Serum urea nitrogen (E) and serum creatinine (F) were tested at the end of treatment. (G), Collagen fiber area was measured based on Masson staining. # and * represent model vs. sham and drugs vs. model, respectively. Student's *t* test was used to compare means between Sham and Model, and one‐way ANOVA was used to compare Model and treated groups (post hoc by Tukey). ^###^ *p* < 0.001, ^####^ *p* < 0.0001. *p < 0.05.

Chronic kidney disease (CKD) is a comprehensive kidney disease with chronic inflammation, oxidative stress, and fibrosis as the main features. Unilateral ureteral ligation can cause inflammation and fibrosis in the kidney, recapitulating the characteristics of CKD in humans. Ureteral obstruction alters renal hemodynamics and metabolic changes, causing excessive deposition of extracellular matrix and renal fibrosis, resulting in damage to essential components such as tubules and glomeruli, which subsequently leads to increased serum urea nitrogen and creatinine. Therefore, we established a UUO mouse model to evaluate the efficacy of CS17919 and GS‐4997 in vivo (Figure 2A). In this model, serum urea nitrogen, creatinine and kidney weight were dramatically increased in the model group compared with the sham group (Figure 2D,E and Figure S1D). After drug intervention, both CS17919 and GS‐4997 significantly reduced serum urea nitrogen (Figure 2D). Furthermore, both CS17919 and GS‐4997 showed a non‐significant tendency to decrease serum creatinine compared with the model group (Figure 2E).

We further examined kidney fibrosis by Masson staining and inflammation and necrosis by H&E staining (Figure 2F). As shown, mice in the model group demonstrated more severe pathological damage, including interstitial fibrosis in the cortical region, necrosis, congestion and hemorrhage in the medullary region, structural disorder of some renal tubules, and diffuse fibrosis. After the intervention, both drugs tended to improve kidney injury such as glomerular swelling and dilatation of renal tubules. Collagen deposition is an essential feature of renal fibrosis and was dramatically increased in the UUO mice compared with the sham group. As expected, both CS17919 and GS‐4997 showed a tendency to reduce the area of kidney fibrosis in the UUO model (Figure 2G). The presence of inflammatory cell infiltration in the renal interstitium of the model group was observed by H&E staining. At the same time, many renal tubular structures were dilated and showed necrosis disappearance, and some of the renal tubules showed a protein tubular pattern. Treatment improved inflammatory infiltration and interstitial fibrosis scores, especially in the CS17919 high‐dose group, which improved significantly (Figure 2B,C). Taken together, CS17919 improved kidney injury and fibrosis with an efficacy equivalent to that of GS‐4997.

### 3.4 CS17919 alleviated glomerular sclerosis in a DKD model

**FIGURE 3:**
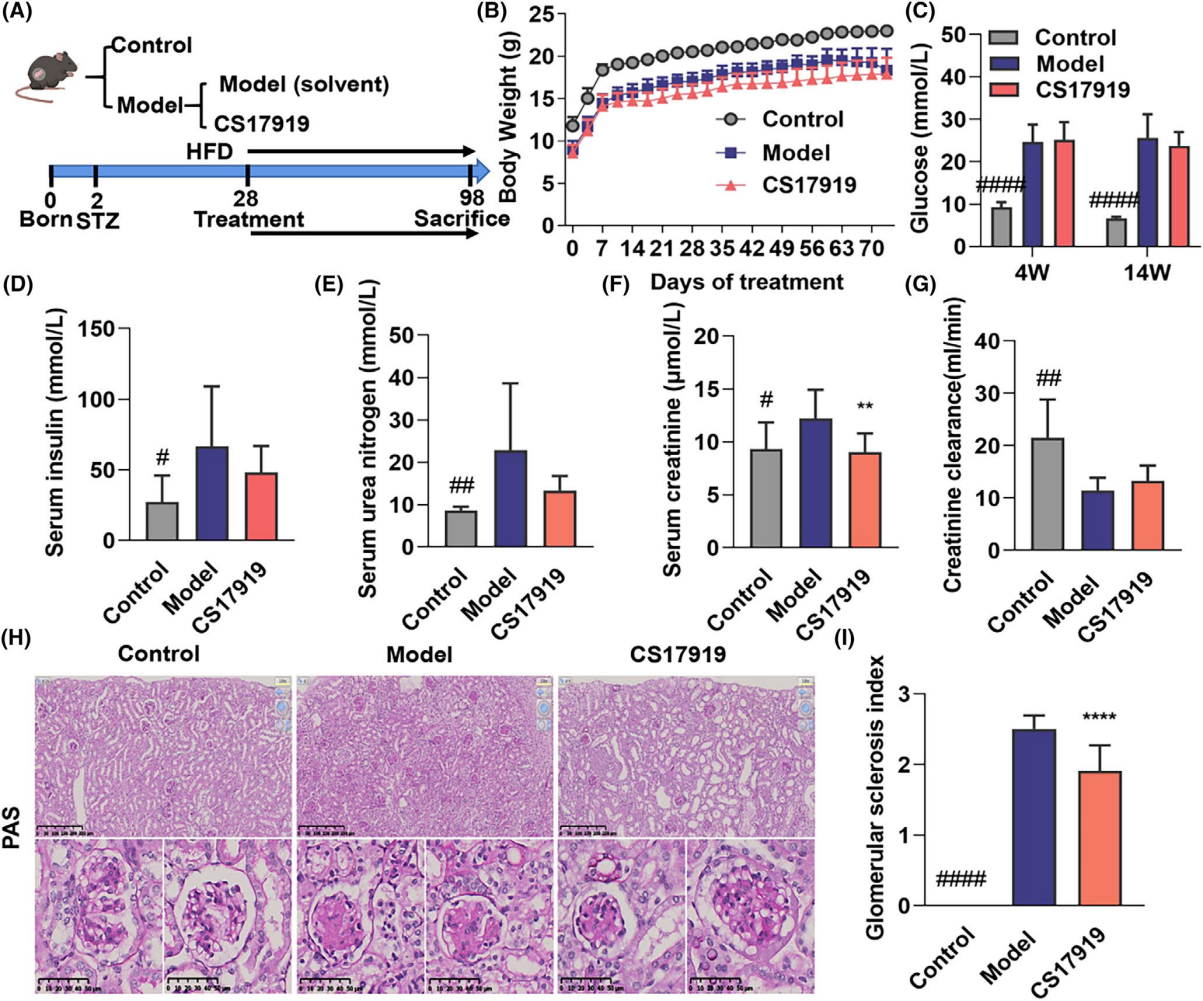
Evaluation of CS17919 in a DKD mouse model. (A), Schematic representation of CS17919 study in the DKD model. (B), Body weight of mice was measured during the treatment. (C), Fasted blood glucose was tested at week 4 (grouping) and week 16 (sacrifice). Serum insulin (D), serum urea nitrogen (E), serum creatinine (F), and creatinine clearance (G) were measured at the end of treatment. (H), Histological changes were assessed by PAS staining. Scale bar: 250 μm. (I), Glomerular sclerosis index (GSI) was obtained based on the PAS staining. *Student's t test was used between groups*. # and * represent model vs. control and CS17919 vs. model, respectively. ^#^ *p* < 0.05, ^##^ *p* < 0.01, ^####^ *p* < 0.0001. **p < 0.01, ****p < 0.0001.

DKD is a type of CKD that results in kidney injury such as glomerular sclerosis due to persistent hyperglycemia. Therefore, we established a STZ+ HFD‐induced DKD model (Figure 3A). Ten weeks of CS17919 administration resulted in slight weight loss along with improved renal coefficients and urine ALB (Figure 3B, Figure S1E,F). Blood glucose was markedly elevated in this model after 4‐weeks of STZ induction and mildly reduced by the CS17919 intervention (Figure 3C). CS17919 also slightly increased insulin sensitivity compared with the model group, indicating its therapeutic potential in diabetes (Figure 3D).

Serum urea nitrogen and creatinine levels were also elevated in this model and highly correlated with the glomerular filtration rate (GFR). CS17919 intervention significantly lowered serum creatinine and showed a non‐significant tendency to reduce serum urea nitrogen (Figure 3E,F). CS17919 also showed a non‐significant tendency to improve creatinine clearance, an index correlated with GFR, which had decreased dramatically in the model group (Figure 3G). In the histopathological study, PAS staining of renal tissues revealed severe glomerular sclerosis in mice in the model group (Figure 3H). CS17919 treatment resulted in a reduction in glomerular sclerosis, mesangial proliferation, and matrix deposition. Furthermore, CS17919 significantly reduced the Kretzler‐created glomerular sclerosis score compared with the model group (Figure 3I). Based on the scoring system, this indicates an improvement in the degree of glomerular sclerosis from severe to moderate. These results show that CS17919 alleviates glomerular sclerosis and improves GFR in DKD.

### 3.5 CS17919 ameliorated liver inflammation and fibrosis in a NASH model

As mentioned in the PK study, CS17919 also accumulates significantly in the liver, suggesting that liver‐related diseases, such as NASH, could be potentially to be treated with CS17919. However, in two phase III clinical trials focusing on treatments for NASH, GS‐4997 failed to meet an acceptable standard, indicating that GS‐4997 alone is insufficient to treat the disease. Therefore, we speculated that a combination of drugs with different mechanisms could be used to treat NASH. In another study, we developed a thyroid hormone receptor‐β agonist CS27109, which showed pronounced efficacy in animal models. We speculated that CS27109 and CS17919 together might act synergistically to provide an effective treatment for NASH by targeting inflammation and fibrosis.

**FIGURE 4:**
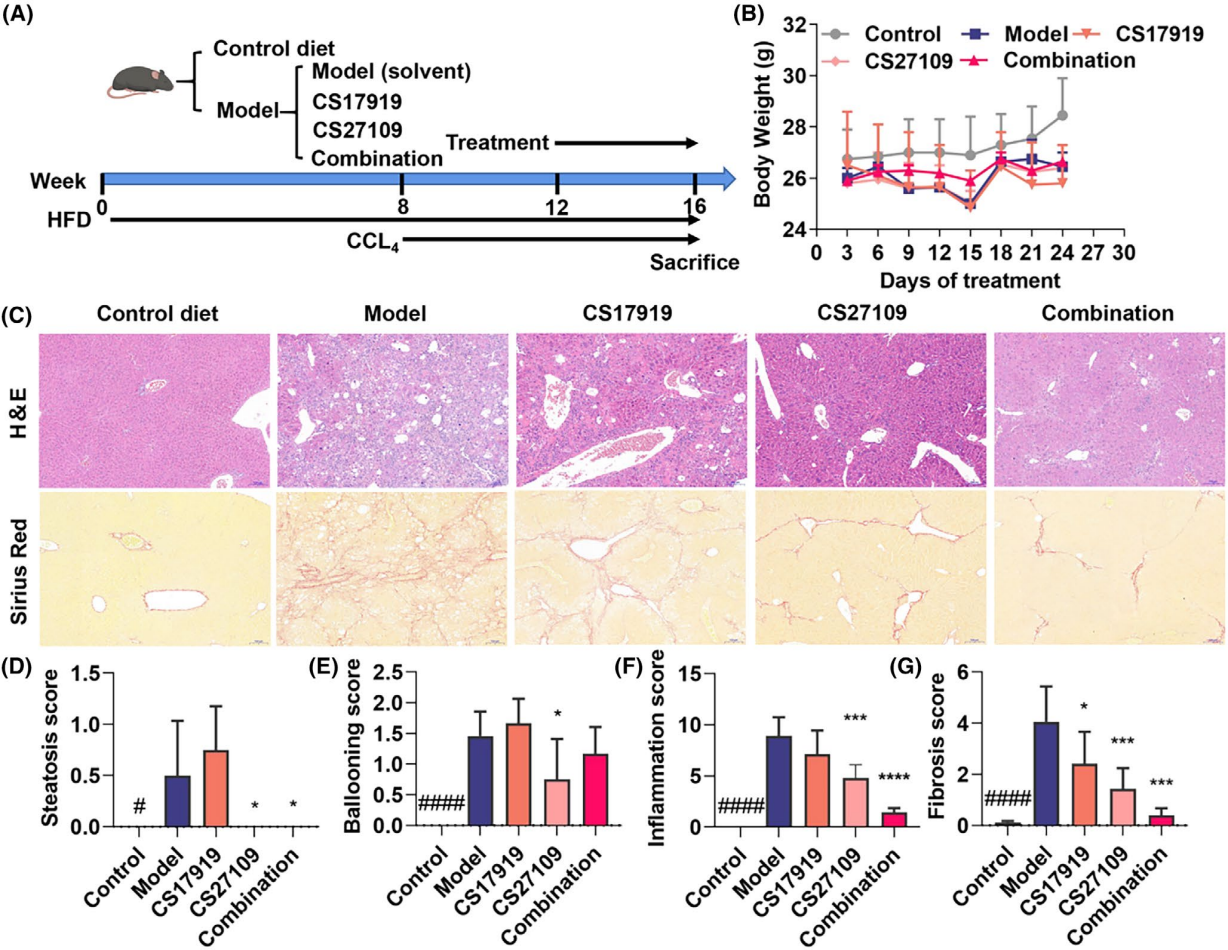
Evaluation of combination of CS17919 and CS27109 in a NASH mouse model. (A), Schematic representation of combination study in the NASH model. (B), Body weight of mice was measured during the treatment. (C), Histological changes were assessed by H&E and Sirius Red staining. Scale bar: 100 μm. Steatosis (D), ballooning (E), and inflammation (F) scores were obtained based on the H&E staining. (G), Liver fibrosis area was determined based on the Sirius Red staining. *Student's t test was used to compare means between Sham and Model, and one‐way ANOVA was used to compare Model and treated groups* (post hoc by Tukey). ^#^ and * represent model vs. sham and drugs vs. model, respectively. ^#^ *p* < 0.05, ^####^ *p* < 0.0001. *p < 0.05, ***p < 0.001, ****p < 0.0001.

To validate this, we established a CCL_4_‐ and HFD‐induced NASH model and tested the efficacy of each agent singly and their combination (Figure 4A). In this model, we found that serum AST, ALT, and LDL‐c increased dramatically, HDL‐c was significantly reduced, and TG and TC were not statistically altered (Table 3). After intervention, CS17919 reduced serum LDL‐c and ALT, tended to lower AST, and improved HDL‐c. CS27109 dramatically reduced serum LDL‐c, ALT, and AST, without affecting other parameters. Interestingly, the combination of both drugs further reduced serum LDL‐c, ALT, and AST and improved HDL‐c.

**TABLE 3:** Blood biochemical parameters of mice after treatment.

Tests	Control diet	Model	CS17919	CS27109	Combination
ALT (U/L)	13.2 ± 2.7^c^	176.1 ± 62.1	120.9 ± 80.3^a^	68.7 ± 18.8^c^	59.5 ± 15.3^c^
AST (U/L)	55.7 ± 14.9^c^	228.8 ± 132.3	193.3 ± 110.1	113.0 ± 36.2^a^	112.7 ± 42.1^a^
LDL‐C (mmol/L)	0.044 ± 0.014^b^	0.119 ± 0.050	0.054 ± 0.028^a^	0.040 ± 0.026^b^	0.036 ± 0.026^b^
HDL‐C (mmol/L)	0.748 ± 0.116^a^	0.560 ± 0.174	0.604 ± 0.231	0.665 ± 0.284	0.853 ± 0.258^a^
TC (mmol/L)	0.840 ± 0.123	0.753 ± 0.203	0.719 ± 0.251	0.785 ± 0.277	0.937 ± 0.303
TG (mmol/L)	0.398 ± 0.239	0.311 ± 0.097	0.223 ± 0.106	0.329 ± 0.341	0.383 ± 0.410

*Note*: a–c indicates significant difference compared with Model group (data were shown as mean ± SD), a: *p* < 0.05, b: *p* < 0.01, c: *p* < 0.001.

Furthermore, we observed that neither drug alone nor the drugs in combination adversely affected the body weight of the mice, indicating that the side effects of the combination are tolerable (Figure 4B). We also found that co‐administration resulted in better improvement in the liver weight of the mice (Figure S1G). We then characterized pathological changes among the different groups by H&E and Sirius Red staining (Figure 4C). This model was characterized by mild steatosis, severe ballooning, inflammation, and fibrosis (Figure 4D–G). After intervention, CS17919 alone significantly reduced the area of liver fibrosis (Figure 4G), tended to improve inflammation (Figure 4F) but barely improved steatosis and ballooning (Figure 4D,E), indicating a mild effect on NASH, which accords with the clinical results. In contrast, CS27109 alone exhibited a pronounced efficacy, improving all pathologic features of NASH, including liver steatosis, inflammation, ballooning and fibrosis. Furthermore, the combination further reduced liver inflammation and fibrosis, showing a synergistic effect (Figure 4F,G). In sum, despite its mild effect as a single agent, CS17919 exerts synergistic effects with CS27109 to ameliorate NASH.

## 4 DISCUSSION

In this study, we developed a new ASK1 inhibitor, CS17919, with pronounced in vitro activity offering cell protection against oxidative stress. CS17919 also exhibited favorable PK properties and had a higher safety profile than the phase III trial ASK1 inhibitor GS‐4997. Finally, we showed that CS17919 alone exerted mild in vivo efficacy in different animal models but showed a robust synergistic effect with CS27109 in the NASH model, indicating its potential in the treatment of metabolic‐related diseases, such as DKD and NASH.

Although GS‐4997 has been tested in multiple clinical trials, it failed in a phase III NASH trial. We analyzed the clinical data and found that targeting ASK1 alone may be insufficient to beat the disease.^25^ In the DKD clinical trial, GS‐4997 did not meet its primary endpoint but appeared to slow the progression of diabetic kidney disease by inhibiting creatinine secretion.^26^ Even though GS‐4997 was not reported to have severe side effects in these clinical trials, it showed high‐dose cytotoxicity in different cell lines in our study, including in L02 cells (a normal human liver cell line), MRC‐5, and HUH7, while in contrast cell viability was not affected by CS17919 (Figure 1B–D). In addition, CS17919 showed promising tolerability in three animal studies without significant reduction in body weight of the animals. Therefore, we hypothesized that CS17919 may have a wider dosing window than GS‐4997, resulting in improved efficacy and fewer side effects.

In the UUO model, ASK1 deletion and inhibition by GS‐444217 (another structurally similar ASK1 inhibitor to GS‐4997) has been shown to attenuate kidney fibrosis by preventing activation of p38 and JNK.^27^ However, GS‐4997 has not been evaluated in this model, but in a non‐diabetic 5/6 nephrectomy (5/6 Nx) rat model,^28^ GS‐4997 alone barely improved CKD‐related characteristics, but preserved kidney function. Therefore, in this study, for the first time, we evaluated both compounds in the UUO model. Our findings confirm that both CS17919 and GS‐4997 improve kidney function by reducing serum urea nitrogen and serum creatinine (Figure 2D,E).

Overactivation of ASK1 has also been observed in DKD patients and animal models,^29^ and GS‐444217 was shown to halt the progression of glomerular sclerosis and improve renal function impairment associated with a reduction in kidney activation of p38, JNK and MAPK.^30^, ^31^, ^32^ Accordingly, we developed an animal model by STZ and HFD induction, showing key characteristics of DKD such as high blood glucose and glomerular sclerosis that agree with the Nos3^−/−^ mouse model, which has been widely used in DKD studies. This model was also characterized by overactivation of JNK,^33^ suggesting it is an appropriate model for evaluating ASK1 inhibitors. Our findings are consistent with those from previous studies for GS‐444217, which showed that both drugs improved glomerular sclerosis, without affecting blood glucose levels and insulin sensitivity. Furthermore, CS17919 significantly reduced serum creatinine but rarely improved GFR, which agrees with the clinical results for GS‐4997. We can therefore conclude that CS17919 has the potential to preserve kidney function and attenuate inflammation such as glomerular sclerosis, but it is unlikely to improve fibrosis in metabolic‐related kidney diseases. Urinary albumin failed to change significantly in this model (data not shown), and further studies are warranted.

As GS‐4997 alone failed to successfully alleviate NASH, several combination therapies have been developed to treat this indication (NCT03449446, NCT02781584, and NCT02466516). However, these drug combinations tend to decrease NASH scores or attenuate liver fibrosis without achieving the primary endpoint. Therefore, we need to develop more drug combinations to effectively improve liver steatosis, inflammation, and fibrosis. The mechanism of NASH is complicated, and lipid and inflammatory pathways play a critical role in disease progression. HFD‐induced lipogenesis is the first stage of NASH, which in turn causes oxidative stress and stimulates inflammation, eventually leading to liver fibrosis and cirrhosis. Consequently, we evaluated the in vivo efficacy of combination therapy using CS17919 and CS27109 for dual targeting of ASK1 and THRβ. CS27109 is a potent THRβ agonist developed by our company, showing favorable oral PK properties and promising preclinical efficacy (unpublished data). It should be noted that MGL‐3196, a phase III THRβ agonist, has been meeting the primary endpoint recently, and is the first drug approved by the FDA for the treatment of NASH.

Interestingly, no preclinical or clinical research has reported using the synergistic effect of a combination of these two targets to treat NASH. Therefore, this is the first study to evaluate this combination therapy in a NASH animal model. In vitro results showed that CS17919 and CS27109 were metabolized slowly in liver microsomes and weakly inhibited CYP1A2, CYP2B6, CYP2C8, CYP2C9, CYP2C19, CYP2D6, and CYP3A4 (Tables S2 and S3). This result suggests that the amelioration of inflammation and fibrosis by the combination of CS17919 and CS27109 does not appear to be achieved by DDI. Interestingly, the combination of CS17919 and CS27109 synergistically improved liver inflammation and fibrosis, which agrees with our hypothesis. CS17919 tends to increase ballooning, but the exact reason is not clear, and the effect of the combination is mainly due to CS27109. This suggests that the drug combination is not more effective than CS27109 alone, but also does not show a significant difference. However, we do not know the reason for this and further studies are needed. Furthermore, we also observed that more than half of the mice in the model group (5/9) had progressed to hepatocellular carcinoma, while the occurrence of carcinoma in the other groups was relatively low: 3/8 for the CS17919 group, 2/8 for the CS27109 group, and notably 0/8 for the combination group (data not shown). The anticancer or cancer preventive effect of both drugs needs to be further confirmed. We conclude that CS17919 alone mildly alleviates liver inflammation and fibrosis but when combined with CS27109 it synergistically improves NASH.

## 5 CONCLUSIONS

In summary, we developed a high‐potency ASK1 inhibitor CS17919 and evaluated its efficacy in CKD and NASH as a single agent or part of a drug combination. CS17919 tended to show an anti‐inflammatory and antifibrotic effect in these models and exhibited a strong synergistic effect with CS27109 in the treatment of NASH. These findings suggest that despite its clinical failure as a single agent, CS17919 remains a potential treatment for these indications when appropriately combined with other targeted therapies.

## AUTHOR CONTRIBUTIONS

Guoqiang Liao, Xuhua Mao and Shengjian Huang planned the experiments, analyzed the data, prepared tables and figures, and finished the draft and manuscript. Shengjian Huang and Zhengli Chen revised the manuscript. Kun Zhang and Yu Zhang conducted in vitro assays and PK studies, respectively. Guoqiang Liao, Xuhua Mao and Yiru Zhao conducted animal experiments. Qianjiao Yang designed and synthesized CS17919. Zhengli Chen and Beizhong Chen carried out pathological experiments and evaluation.

## FUNDING INFORMATION

The authors declare that no funds, grants, or other support were received during the preparation of this manuscript.

## CONFLICT OF INTEREST STATEMENT

The authors have no relevant financial or non‐financial interests to disclose.

## ETHICS STATEMENT

All animal experiments were performed according to the guidelines for the care and use of animals approved by Chengdu Chipscreen Pharmaceutical Ltd. Council.

## Supporting information


Data S1.
