# Supplementary material for: Targeting ASK1 by CS17919 alleviates kidney‐ and liver‐related diseases in murine models
Source: Animal Model Exp Med. 2024 Jun 14;8(1):102–13. doi: 10.1002/ame2.12437 (PMC11798738; doi:10.1002/ame2.12437)
Supplement: Supplementary file 1 — Data S1. [file AME2-8-102-s001.docx]

**Table S1.** Enzyme activity analysis working solution preparation and analysis procedure.

| **Buffers preparation** | | |
| --- | --- | --- |
| Kinase buffer = 1Xsupplemented  enzymatic buffer | Dilute 1 volume of enzymatic buffer 5× with 4 volumes of distilled water and complement it with all the supplements required by the kinase of interest, i.e. DTT, MgCl2, CaCl2, and Calmodulin, etc. | |
| **Reagents preparation** | | |
| STK Substrate(1),(2),(3)-biotin  Final concentration: 625 nmol/L | 1. Reconstitute with distilled water to obtain a 50 µmol/L STK Substrate stock solution (refer to product label).  2. Dilute the 50 µmol/L STK Substrate stock solution with 1× kinase buffer to prepare a working solution which has 5× the required final concentration for the enzymatic step (10 µL). | |
| Streptavidin-XL665  Final concentration: 100 nmol/L | 1. Reconstitute with distilled water to obtain a 16.67 µmol/L Streptavidin stock solution (refer to product label).  2. Dilute the 16.67 µmol/L Streptavidin stock solution with detection buffer to prepare a working solution which has 4× the required final concentration for the final volume assay (20 µL).  E.g. For 125 nmol/L final concentration prepare a 500 nmol/L working solution: dilute 33.3-fold the reconstituted 16.67 µmol/L Sreptavidin-XL665 stock solution. | |
| STK Antibody-Cryptate  Kit for 1000 tests | Reconstitute the vial with 5 mL of Detection buffer to get the ready to use STK-antibody-cryptate solution | |
| Compounds | Dilute compound stock solution with kinase buffer to prepare a working solution (10 µL). | |
| Kinase  Final concentration: 300 nmol/L | Dilute the kinase stock solution with kinase buffer to prepare a working solution which has 5× the required final concentration for the enzymatic step (10 µL). | |
| ATP | Dilute the ATP stock solution (5 mmol/L) with kinase buffer to prepare a working solution which has 5× the required final concentration for the enzymatic step (10 µL). E.g. For an ATP 100 µmol/L in the enzymatic step, prepare a 500 µmol/L ATP working solution. | |
| **Assay protocol** | | |
| **Kinase assay** | | **Negative** |
| Enzymatic step — 10 µL | | |
| 4 µL of compounds*  2 µL of STK Substrate (1),(2),(3)-biotin  2 µL of kinase  2 µL of ATP** | | 4 µL of compounds*  2 µL of STK Substrate (1),(2),(3)-biotin  2 µL of kinase buffer  2 µL of ATP** |
| Seal the plate and incubate*** at room temperature for 1h. | | |
| Detection step — 10 µL | | |
| 5 µL of Streptavidin-XL665  5 µL of STK Antibody-cryptate | | |
| Seal the plate and incubate 1 hour at room temperature. | | |
| Remove the plate sealer and read the values at wavelengths 665 nm and 620 nm with an EnVision plate reader. | | |
| * For low volume compound addition, adjust volume to 4 µL with 1x kinase buffer. Keep DMSO ≤2% in the enzymatic step.  ** The kinase reaction is started by the addition of ATP (Enzymatic step) and is stopped by the addition of the detection.  reagents which contain EDTA (detection step). The incubation time is dependent on the activity of the kinase tested.  *** The incubation period for the enzymatic step is optimized depending on the kinase. | | |

**Table S2.** Metabolic studies of **CS17919** and **CS27109** on CYP enzymes *in vitro.*

| CYP inhibition | CYP  1A2 | CYP  2B6 | CYP  2C8 | CYP  2C9 | CYP  2C19 | CYP  2D6 | CYP3A4 (Midazolam) | CYP3A4 (Testosterone) |
| --- | --- | --- | --- | --- | --- | --- | --- | --- |
| CS17919 IC_50_ (μmol/L) | ＞10 | ＞10 | ＞10 | ＞10 | ＞10 | ＞10 | 3.02 | ＞10 |
| CS27109  IC_50_ (μmol/L) | ＞10 | ＞10 | ＞10 | 5.10 | ＞10 | ＞10 | ＞10 | ＞10 |

**Table S3.** Metabolic studies of **CS17919** and **CS27109** on liver microsomes *in vitro*.

|  | CS17919 | | CS27109 | |
| --- | --- | --- | --- | --- |
| Species | T_1/2_ (min) | Cl_int_ (mL/min/kg) | T_1/2_ (min) | Cl_int_ (mL/min/kg) |
| Human | ＞145 | 8.6 | ＞120 | 0.83 |
| Rat | ＞145 | 17.3 | ＞120 | 4.24 |
| Mouse | ＞145 | 38 | ＞120 | 7.72 |


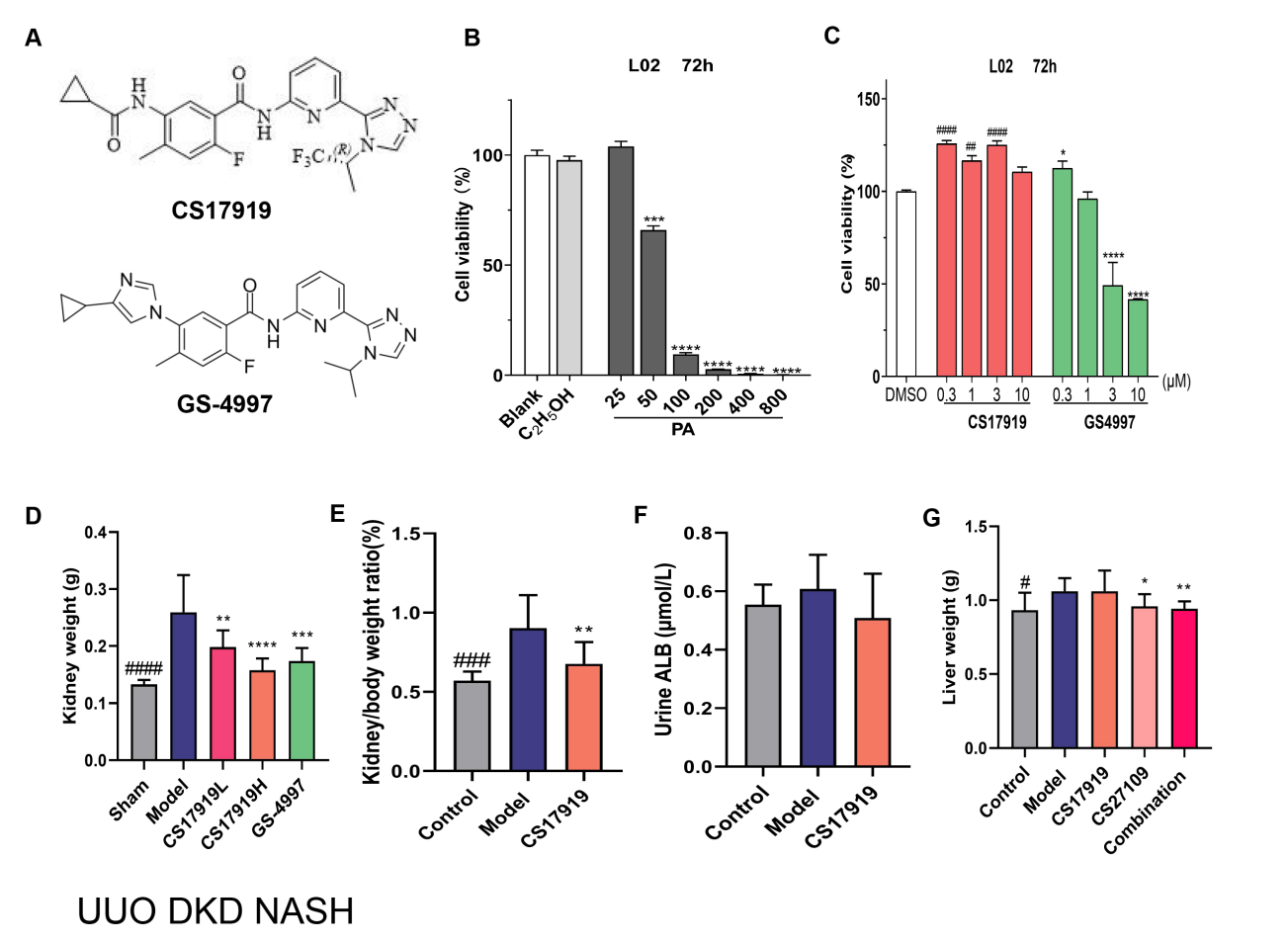


**FIGURE S1** (A) Chemical structural formulae of **CS17919** and **GS-4997**. (B) Viability of L02 cells 72 h after 25–800 μmol/L (2-fold dilution) PA stimulation. (C) The effects of **CS17919** and **GS-4997** at different doses (0.3–10 μmol/L) on L02 cells. (D) Kidney weights in the UUO model. (E) Renal coefficients in the DKD model. (F) Urine ALB in the DKD model. (G) Liver weights in the NASH model. *: PA *vs.* Blank; **GS-4997** *vs.* DMSO; Treatment *vs*. Model. **#**: **CS17919** *vs*. DMSO; Control *vs*. Model. * *p* < 0.05, **##** *p* < 0.01, *** *p* < 0.001, **####** and **** *p* < 0.0001 were obtained by one-way ANOVA.
